# Integrating rare genetic variants into *DPYD* pharmacogenetic testing may help preventing fluoropyrimidine-induced toxicity

**DOI:** 10.1038/s41397-023-00322-x

**Published:** 2024-01-12

**Authors:** Romain Larrue, Sandy Fellah, Benjamin Hennart, Naoual Sabaouni, Nihad Boukrout, Cynthia Van der Hauwaert, Clément Delage, Meyling Cheok, Michaël Perrais, Christelle Cauffiez, Delphine Allorge, Nicolas Pottier

**Affiliations:** 1 Univ. Lille, CNRS, Inserm, CHU Lille, Institut Pasteur de Lille, UMR9020-U1277-CANTHER-Cancer Heterogeneity Plasticity and Resistance to Therapies, F-59000 Lille, France; 2 Service de Toxicologie et Génopathies, CHU Lille, F-59000 Lille, France

**Keywords:** Predictive markers, Genetic testing

## Abstract

Variability in genes involved in drug pharmacokinetics or drug response can be responsible for suboptimal treatment efficacy or predispose to adverse drug reactions. In addition to common genetic variations, large-scale sequencing studies have uncovered multiple rare genetic variants predicted to cause functional alterations in genes encoding proteins implicated in drug metabolism, transport and response. To understand the functional importance of rare genetic variants in *DPYD*, a pharmacogene whose alterations can cause severe toxicity in patients exposed to fluoropyrimidine-based regimens, massively parallel sequencing of the exonic regions and flanking splice junctions of the *DPYD* gene was performed in a series of nearly 3000 patients categorized according to pre-emptive DPD enzyme activity using the dihydrouracil/uracil ([UH_2_]/[U]) plasma ratio as a surrogate marker of DPD activity. Our results underscore the importance of integrating next-generation sequencing-based pharmacogenomic interpretation into clinical decision making to minimize fluoropyrimidine-based chemotherapy toxicity without altering treatment efficacy.

## Introduction

An increasing number of clinically relevant association between drug response and genomic variation has been reported over the past years, resulting in evidence-based pharmacogenetic guidelines [1, 2]. For instance, the Pharmacogenomics Knowledge base PharmGKB (https://www.pharmgkb.org) has collected and curated information for more than 740 drugs and, to date, contains 189 clinical guidelines and 868 drug label annotations approved by various pharmaceutical regulatory organizations such as the US Food and Drug Administration (FDA) or the European Medicines Agency (EMA). Nevertheless, although many patients would benefit from pharmacogenetics-based prescription policy [3], only limited applications are observed in clinical practice, especially in primary care [4–7]. Indeed, genetic testing is in most cases performed retrospectively when adverse side effects arise or when a drug lacks efficacy. Main barriers to the implementation of pharmacogenetics into routine clinical practice are the lack of awareness and education of physicians and pharmacists, solid scientific evidence of pharmacogenomic biomarkers, harmonized and implementable pharmacogenomic guidelines and in some instances, the absence of a dedicated infra-structure to integrate pharmacogenetics testing into the workflow of health care providers [6, 8]. Seminal studies have notably shown the importance of common genetic variants affecting phase I or phase II enzymes in the resistance to various pharmacological agents or the occurrence of life-threatening side effects [9]. Prominent examples include the association between common defective TPMT alleles and the risk of hematotoxicity following 6-mercaptopurine exposure [10] or the impact of frequent specific CYP2C19 polymorphisms on clopidogrel efficacy [11]. Nevertheless, these common genetic variants, while important, only account for little of the inherited individual variation in drug response and a substantial fraction of the genetically encoded variability in drug pharmacokinetics remains to be elucidated. Interestingly, recent large-scale studies have unveiled that more than 90% of the genetic variability in genes associated with drug metabolism and disposition is assigned to rare genetic variants, but the functional impact of such rare pharmacogenetic variants on drug response remains poorly documented.

Fluoropyrimidine-based treatment regimens are the standard therapy for many distinct types of advanced solid tumors including breast, colorectal as well as head and neck cancers [12]. Nevertheless, up to 30% of patients will experience serious adverse drug reactions such as diarrhea, stomatitis, mucositis, myelosuppression or neurotoxicity, which can be lethal in 0.5–1% of cases [12, 13]. Dihydropyrimidine dehydrogenase (DPD), the initial and rate limiting enzyme involved in the catabolism of 5-fluorouracil (5-FU), is responsible for the elimination of 80–85% of the administered dose. Plasma concentrations of uracil ([U]), the endogenous substrate for DPD, or its product dihydrouracil (UH_2_) are routinely used as a surrogate marker for systemic DPD activity [14]. Indeed, pretreatment [U] and [UH_2_]/[U] ratio are highly correlated with systemic DPD activity and many studies have shown a relationship between fluoropyrimidine-induced toxicity and a DPD phenotype characterized by high [U] or low [UH_2_]/[U] ratio [14, 15]. However, the equipment required as well as the recommended pre-analytical conditions for the measurement of [U] and [UH_2_] are not widely available in many clinical laboratories [16, 17]. Therefore, implementation of alternative approaches such as *DPYD*-based pharmacogenetic assays are convenient complementary methods to accurately predict DPD activity [16]. Indeed, according to PharmGKB, more than 20 loss-of-function *DPYD* variants have been reported to alter DPD enzymatic activity, and consequently patients harboring such variants are exposed to an increased risk of severe toxicity when receiving standard dose of fluoropyrimidine. For this reason, international guidelines now recommend pre-emptive *DPYD* genotyping for several clinically relevant defective variants: i.e., c.1905+1G>A (DPYD*2A), c.1679T>G (DPYD*13), c.2846A>T, and Haplotype B3 (c.1236G>A or c.1129–5923C>G) as well as genotype-guided prescribing recommendations [17, 18].

In this study, using Next Generation Sequencing (NGS), we comprehensively assessed the relationship between *DPYD* genotype and DPD phenotype in a series of 2 972 patients and identified new rare clinically relevant variants associated with DPD deficiency. Our results also show that rare *DPYD* genetic variants account for a significative part of the interindividual variability of DPD activity. Therefore, comprehensive NGS-based genotyping instead of candidate SNP interrogation should be considered for the guidance of personalized fluropyrimidine therapy.

## Materials and methods

### Studied cohort

All patients included in this study were eligible for an uracil analog-based chemotherapy (Supplementary Table S1). Only those for which both *DPYD* genotype and DPD phenotype were available were included. The protocol has been certified to be in accordance with French laws by the Institutional Review Board of Centre Hospitalier Universitaire de Lille (France). Genotyping analysis and DPD phenotyping were performed as described in our local regular protocol to identify DPD-deficient patients at increased risk of severe fluoropyrimidine-induced toxicity. However, information regarding fluoropyrimidine toxicity was not available. All patients provided their written informed consent for genetic analysis and to publish this paper in accordance with institutional guidelines and the Declaration of Helsinki and Istanbul. The DNA collection was registered by the Ministère de l’Enseignement Supérieur et de la Recherche (Paris, France) under the number: DC-2008–642.

### DPD phenotyping

Pretreatment Plasma Uracil [U] and dihydrouracil [UH_2_] were quantified using a Waters TQD UPLC^®^-MS/MS System (Waters Corp., Milford, MA, USA) equipped with an electrospray ionization interface according to the method described by Coudore et al. [19]. Data acquisition and processing were performed using MassLynx v.4.0 software. DPD activity was categorized as normal, partial or complete deficiency based on previous reports using the [UH_2_]/[U] ratio [20–26]. Indeed, although no consensual cut-off values for the [UH_2_]/[U] ratio has been established yet, a [UH_2_]/[U] ratio cut-off below or equal to 10 was chosen for DPD deficiency as it has been previously demonstrated as a good predictor of fluoropyrimidine toxicity [15, 27]. Therefore, partial DPD deficiency was defined as [UH_2_]/[U] ≤ 10 whereas complete DPD deficiency was defined as [UH_2_]/[U] ≤ 1. Alternatively, DPD activity can also be estimated by measuring [U] and a cut-off value over or equal to 16 µg/mL is used to define partial deficiency and over 150 µg/mL for complete deficiency [15].

### *DPYD* genotyping

All patients gave their written informed consent for genetic testing. Genomic DNA was extracted from peripheral blood using Chemagic Star (Chemagen, Baesweiler, Germany) and then quantified using the NanoDrop® spectrophotometer (ThermoFisher Scientific, Waltham, MA, USA) according to the manufacturer’s instructions. Genomic sequence of the *DPYD* gene was retrieved from the NCBI website and the Reference Sequence NG_008807.2 was subsequently used. Primers were designed to include all exonic regions and at least 30 bp of each flanking intron using Fluidigm D3™ assay design web-based tool. A total of 64 unique primer pairs were created and are listed in Supplementary Table S2. Custom-designed primer pairs to target *DPYD* exonic regions and exon–intron boundaries were designed and optimized for the Fluidigm Access Array (Fluidigm, South San Francisco, CA, USA). Amplification of genomic DNA was performed in up to 10-plex PCR reaction wells, followed by addition of barcode indexes and sequencing adaptors by further PCR according to manufacturer’s instructions. Pooled amplicons were harvested and diluted to prepare unidirectional libraries for 150 base-pair (bp) paired-end sequencing on Illumina MiSeq sequencing platform (Illumina, San Diego, CA, USA). Illumina NGS reads were trimmed for base Phred quality control (mean quality in a 30 bp sliding window >20 and 3′ base quality ≥6) and aligned with Burrows–Wheeler Aligner (v0.6.1-r112-master) on hg19 human genome reference sequence. Variant-calling was achieved using MiSeq Reporter v2.6, GATK v3.7 or GATK v4.1.4.0 (Genome Analysis Toolkit) [28] without downsampling or removal of PCR duplicates; variants with quality/depth < 5 or depth < 30 were filtered. All very rare (MAF ≤ 0.1%) and novel variants identified by NGS analysis were validated by Sanger sequencing (Table S2). The functional consequences of each variant were estimated by in silico analysis, using bioinformatic prediction tools such as SIFT, PolyPhen-2 or CADD and on the basis of the ACMG classification.

### Statistical analyses

Sample size was chosen empirically based on our previous experiences in the calculation of experimental variability; no statistical method was used to predetermine sample size and no samples or data points were excluded from the reported analyses. Data are described as the medians ± standard deviations, or *n* (%). Since [U] and [UH_2_]/[U] values were not normally distributed, non-parametric tests were performed. Allelic frequencies and genotype distribution were estimated by gene counting and tested for Hardy–Weinberg equilibrium. For the comparison of proportions and to evaluate the Hardy–Weinberg equilibrium, we used the chi-square test. As in most cases, a low number of individuals carries the alternate allele homozygote, the influence of the genotypes on DPD activity was assessed by clustering genotypes into a dominant inheritance model. Then, genotypes were compared using non parametric Mann–Whitney and Kruskal–Wallis tests. The level of significance was set at *p* < 0.05. All analyses were two-sided. Statistical analyses were performed using Prism® 5.0 (GraphPad) and JMP (SAS) software.

## Results

### Inter-individual variability of pretherapeutic DPD enzyme activity

This retrospective study included 2972 subjects. Mean patient age was 65 ± 11 years, and the sex ratio (M/F) was 1.2 (Supplementary Table S1). Using a cut-off value below or equal to 10 for the [UH_2_]/[U] ratio, 580 patients (19.7%) were categorized with partial DPD deficiency, whereas no patient exhibited complete DPD deficiency. Mean age did not significantly differ between the partial DPD deficiency group and the normal DPD group (Supplementary Table S1). Overall, [U] and [UH_2_]/[U] values identified 628 patients (21.1%) with DPD deficiency, but these parameters were in agreement in only 114 (18.2%) patients (Table 1). Indeed, 466 (15.7%) patients presented [UH_2_]/[U] ≤ 10 and [U] < 16 ng/mL, and 48 (1.6%) presented [UH_2_]/[U] > 10 and [U] ≥ 16 ng/mL (Table 1). The [UH_2_]/[U] level below which [U] values were all ≥ 16 ng/mL was 4.6, and the [U] level above which [UH_2_]/[U] values were all ≤ 10 was 49 ng/mL, suggesting that a better agreement between [UH_2_]/[U] and [U] values to identify DPD deficiency would require the use of more restrictive thresholds. Based on these results, the current cut-off values for [U] and [UH_2_]/[U] do not identify DPD deficiency in an equivalent manner, and a [UH_2_]/[U] ratio ≤ 10 yields a higher proportion of individuals classified with partial DPD deficiency than [U] levels > 16 ng/mL.

**Table 1:** Number of patients according to the uracil plasma concentration ([U]) and the dihydrouracil/uracil ([UH_2_]/[U]) plasma ratio.

Phenotype	[U] < 16 ng/mL	[U] ≥ 16 ng/mL
[UH_2_]/[U] > 10	2344	48
[UH_2_]/[U] ≤ 10	466	114

### Genetic variants identified in *DPYD*

The group of patients with partial DPD deficiency represented a total of 580 patients, including 134 wild-type patients (DPYD*1/*1) and 446 patients harboring at least one genetic variant (208 patients carried one genetic variant and 238 patients more than one). Overall, genetic variants identified in patients with partial DPD deficiency represent a total of 809 variants. The remaining 2392 patients exhibiting normal DPD activity include 623 wild-type patients (DPYD*1/*1) and 1769 mutated patients in which a total of 3183 genetic variants were identified (831 carrying a single genetic variant and 938 carrying more than one). The mean coverage (read depth) of the identified genetic variants was 1130 (range: 33–4995) for the group of patients with DPD partial deficiency and 1131 (range: 33–7612) for group of patients whose phenotype was unaltered. 30 distinct genetic variants were identified in the group of patients exhibiting partial DPD deficiency (29 single nucleotide polymorphisms and one indel). Among these genetic variants, 23% (7/30) were common (MAF ≥ 1%) and 77% (23/30) were considered as rare /very rare or novel (MAF < 1%), and among these, 58% (13/23) were classified as deleterious according to variant effect prediction algorithms (Table 2). In addition, the majority of variants were missense (77%; 23/30), one was non-sense, one was categorized as indel and two were located in canonical splice sites. Among the remaining variants, 10% (3/30) were synonymous. In the group of patients exhibiting a normal DPD phenotype, 58 unique genetic variants were identified including 56 single nucleotide polymorphisms and two indel. 12% (7/58) were common whereas 88% (51/58) were considered as rare/very rare or novel (MAF < 1%) including 35% (18/51) classified as deleterious by functional prediction algorithms. In addition, the majority of variants were missense (55%, 32/58), two were non sense and six were located in canonical splice sites. Among the remaining variants, 29% (17/58) were synonymous and 2% (1/58) were located in the UTR (Untranslated Regions). All rare genetic variants were heterozygous. Hardy–Weinberg equilibrium for each common and rare variant and allelic frequencies are reported in Supplementary Table S3. As the French law of information and freedom prohibits to collect information on ethnicity, it was thus impossible to provide data frequency according to patient ancestry. We thus made the assumption that our population was mainly European (Supplementary Table S3).

**Table 2:** List of the genetic variants identified in *DPYD* by next generation sequencing.

Position (GRCh37)	Ref	Alt	Statut	Number of patients		Allele Freq.	HGVS		dbSNP ID	Allele	Transcript consequences	SIFT (score)	PolyPhen (score)	CADD(score)	ClinVar ID	GnomAD (Eur. MAF)
				Total	Deficient		Coding DNA	Protein								
***MAF*** ≥ ***1%***																
chr1:97770920	C	T	Het	293	81 (28%)	5.10%	c.2194G>A	p.Val732Ile	rs1801160	*6	missense variant	T (0.1)	B (0.30)	24.4	100080	4.53%
			Hom	5	1 (20%)											
chr1:97915624	A	G	Het	208	32 (15%)	3.63%	c.1896T>C	p.Phe632Phe	rs17376848		synonymous variant			3.576	100088	5.04%
			Hom	4	0 (0%)											
chr1:97981395	T	C	Het	951	178 (19%)	20.24%	c.1627A>G	p.Ile543Val	rs1801159	*5	missense variant	D (0.04)	B (0)	15.46	100092	19.52%
			Hom	126	15 (12%)											
chr1:97981421	C	T	Het	136	31 (23%)	2.36%	c.1601G>A	p.Ser534Asn	rs1801158	*4	missense variant	D (0.01)	B (0.03)	22.8	100094	1.43%
			Hom	2	2 (100%)											
chr1:98039419	C	T	Het	97	19 (20%)	1.67%	c.1236G>A	p.Glu412Glu	rs56038477	hapB3	synonymous variant			9.659	100100	1.40%
			Hom	1	0 (0%)											
chr1:98165091	T	C	Het	486	113 (23%)	8.92%	c.496A>G	p.Met166Val	rs2297595		missense variant	D (0)	PD (0.99)	24.8	100116	8.59%
			Hom	22	2 (9%)											
chr1:98348885	A	G	Het	930	180 (19%)	22.98%	c.85T>C	p.Cys29Arg	rs1801265	*9A	missense variant	T (1)	B (0)		435	22.45%
			Hom	218	40 (18%)											
***1%*** > ***MAF*** ≥ ***0. 1%***																
chr1:97544543	G	T	Het	5	3 (60%)	0.08%	c.3067C>A	p.Pro1023Thr	rs114096998		missense variant	D (0)	B (0.42)	18.94	100069	0.36%
chr1:97547947	T	A	Het	22	15 (68%)	0.37%	c.2846A>T	p.Asp949Val	rs67376798		missense variant	D (0)	PD (0.52)	25.3	88974	0.29%
chr1:97915614	C	T	Het	12	8 (67%)	0.20%	c.1905+1G>A	p.(?)	rs3918290	*2A	splice donor variant			33	432	0.57%
chr1:97981343	A	C	Het	11	6 (55%)	0.19%	c.1679T>G	p.Ile560Ser	rs55886062	*13	missense variant	D (0)	PD (0.94)	27.9	88975	0.031%
chr1:98015269	G	A	Het	7	0 (0%)	0.12%	c.1371C>T	p.Asn457Asn	rs57918000		synonymous variant			11.57	100097	0.24%
chr1:98039437	C	T	Het	5	2 (40%)	0.08%	c.1218G>A	p.Met406Ile	rs61622928		missense variant	T (0.31)	B (0)	19.85	100101	0.67%
chr1:98144726	T	C	Het	8	2 (25%)	0.13%	c.775A>G	p.Lys259Glu	rs45589337		missense variant	D (0)	PD (0.62)	23	235464	0.61%
***MAF*** ≤ ***0.1%***																
chr1:97544541	C	T	Het	1	0	0.02%	c.3069G>A	p.Pro1023Pro	rs749122978		synonymous variant	D (0)	B (0.42)	0.179	NA	0.002%
chr1:97547907	G	A	Het	1	0	0.02%	c.2886C>T	p.Thr962Thr	rs368617815		synonymous variant			11.25	NA	0.006%
chr1:97547921	T	C	Het	3	3 (100%)	0.05%	c.2872A>G	p.Lys958Glu	rs141044036		missense variant	D (0.01)	PD (1)	28.9	551659	0.002%
chr1:97564044	C	T	Het	1	0	0.02%	c.2766+1G>A	p.(?)	rs1355754530		splice donor variant			34	NA	0.001%
chr1:97564177	A	C	Het	1	0	0.02%	c.2634T>G	p.Ser878Arg	rs919596571		missense variant	T (0.36)	B (0.00)	23	874134	0.001%
chr1:97658667	CT	C	Het	1	0	0.02%	c.2579del	p.Gln860Argfs*9	rs746991079		frameshift variant			34	551707	0.004%
chr1:97658736	C	A	Het	1	0	0.02%	c.2511G>T	p.Leu837Leu	rs763174477		synonymous variant			9.956	NA	0.016%
chr1:97700416	C	T	Het	1	0	0.02%	c.2434G>A	p.Val812Ile	rs371313778		missense variant	T (0.11)	B (0.03)	22.9	NA	0.012%
chr1:97700495	C	T	Het	1	0	0.02%	c.2355G>A	p.Leu785Leu	NA		synonymous variant			10.03	NA	
chr1:97700520	G	T	Het	1	1	0.02%	c.2330C>A	p.Ala777Asp	rs374825099		missense variant	T (0.05)	B (0.4)	25.5	NA	0.003%
chr1:97700547	G	T	Het	1	0	0.02%	c.2303C>A	p.Thr768Lys	rs56005131		missense variant	T (0.16)	B (0.05)	22.7	287480	0.019%
chr1:97771751	C	T	Het	3	1 (33%)	0.05%	c.2161G>A	p.Ala721Thr	rs145548112		missense variant	D (0)	PD (1)	31	100082	0.015%
chr1:97771760	C	A	Het	1	1	0.02%	c.2152G>T	p.Val718Leu	NA		missense variant	T (0.24)	B (0.01)	21.3	NA	
chr1:97771837	C	T	Het	1	0	0.02%	c.2075G>A	p.Arg692Gln	rs375436137		missense variant	T (0.09)	B (0.04)	26.8	2412214	0.002%
chr1:97771841	C	T	Het	1	0	0.02%	c.2071G>T	p.Val691Leu	rs202212118		missense variant	T (0.1)	PD (0.97)	29.5	298286	0.015%
chr1:97839112	C	T	Het	1	0	0.02%	c.2058+5G>A	p.(?)	rs367623519		splice region variant			22.7	NA	0.005%
chr1:97847973	C	T	Het	1	1	0.02%	c.1950G>A	p.Trp650*	NA		stop gain			45	NA	
chr1:97915615	G	C	Het	1	0	0.02%	c.1905C>G	p.Asn635Lys	rs3918289		missense variant	T (1)	B (0.02)	1.764	NA	0.002%
chr1:97915674	T	G	Het	1	1	0.02%	c.1846A>C	p.Lys616Gln	rs368146607		missense variant	D (0.01)	PD (0.96)	26.2	NA	0.003%
chr1:97915692	T	C	Het	1	0	0.02%	c.1828A>G	p.Ile610Val	NA		missense variant	D (0.01)	PD (0.85)	24.9	NA	
chr1:97915724	A	G	Het	1	1	0.02%	c.1796T>C	p.Met599Thr	rs147601618		missense variant	T (0.42)	B (0)	18.83	550673	0.006%
chr1:97915745	C	T	Het	1	1	0.02%	c.1775G>A	p.Arg592Gln	rs138616379		missense variant	D (0)	PD (0.97)	29.8	554703	0.002%
chr1:97915769	G	A	Het	1	0	0.02%	c.1751C>T	p.Thr584Ile	NA		missense variant	T (0.12)	B (0.26)	25.2	NA	
chr1:97915777	G	A	Het	1	1	0.02%	c.1743C>T	p.Asp581Asp	rs555178721		splice region variant			11.55	NA	0.005%
chr1:97981321	T	A	Het	2	0	0.03%	c.1701A>T	p.Gly567Gly	rs148372305		synonymous variant			9.725	738289	0.024%
chr1:97981377	C	G	Het	1	0	0.02%	c.1645G>C	p.Ala549Pro	rs140039091		missense variant	D (0)	PD (1)	29.5	NA	<0.001%
chr1:97981407	C	T	Het	1	1	0.02%	c.1615G>A	p.Gly539Arg	rs142619737		missense variant	D (0)	PD (0.97)	23.3	100093	0.020%
chr1:97981408	G	A	Het	1	0	0.02%	c.1614C>T	p.Ala538Ala	rs760853559		synonymous variant			6.617	298288	0.006%
chr1:98015121	C	T	Het	1	0	0.02%	c.1519G>A	p.Val507Ile	rs138391898		missense variant	T (0.93)	B (0)	0.92	NA	0.001%
chr1:98015142	A	G	Het	1	0	0.02%	c.1498T>C	p.Ser500Pro	NA		missense variant	D (0)	PD (0.91)	25.3	NA	
chr1:98015280	T	C	Het	1	1	0.02%	c.1360A>G	p.Ile454Val	rs927463053		missense variant	T (0.71)	B (0)	15.44	NA	0.001%
chr1:98015291	G	C	Het	1	0	0.02%	c.1349C>G	p.Ala450Gly	rs72975710		missense variant	D (0.01)	B (0.07)	28	298294	0.002%
chr1:98039515	A	G	Het	2	0	0.03%	c.1140T>C	p.Ala380Ala	rs150759598		synonymous variant			9.718	NA	0.005%
chr1:98058790	C	T	Het	1	0	0.02%	c.1112G>A	p.Arg371Lys	NA		missense variant	D (0.03)	B (0.07)	25.3	NA	
chr1:98058813	G	A	Het	1	1	0.02%	c.1089C>T	p.Phe363Phe	rs764173823		synonymous variant			13.76	NA	0.002%
chr1:98058829	C	T	Het	1	0	0.02%	c.1073G>A	p.Arg358His	rs573299212		missense variant	D (0)	PD (0.98)	29.9	NA	0.002%
chr1:98058915	T	C	Het	1	0	0.02%	c.987A>G	p.Pro329Pro	NA		synonymous variant			11.46	NA	
chr1:98060643	C	T	Het	1	0	0.02%	c.930G>A	p.Leu310Leu	NA		synonymous variant			11	NA	
chr1:98060721	A	C	Het	1	0	0.02%	c.852T>G	p.Gly284Gly	NA		splice region variant			14.32	NA	
chr1:98144657	C	T	Het	1	0	0.02%	c.844G>A	p.Gly282Arg	NA		missense variant	D (0)	PD (1)	32	NA	
chr1:98164964	C	A	Het	1	1	0.02%	c.623G>T	p.Arg208Leu	rs376073289		missense variant	D (0)	PD (1)	29.6	806173	0.002%
chr1:98164975	G	C	Het	1	0	0.02%	c.612C>G	p.Ser204Ser	rs768519000		synonymous variant			7.559	NA	<0.001%
chr1:98164986	T	G	Het	1	1	0.02%	c.601A>C	p.Ser201Arg	rs72549308		missense variant	D (0)	PD (1)	26.4	NA	0.003%
chr1:98164996	A	G	Het	1	0	0.02%	c.591T>C	p.Pro197Pro	rs758927521		synonymous variant			11.97	1750509	<0.001%
chr1:98165030	T	C	Het	1	0	0.02%	c.557A>G	p.Tyr186Cys	rs115232898		missense variant	D (0, 0.1)	PD (0.98)	25.5	100113	0.21%
chr1:98165042	A	T	Het	1	0	0.02%	c.545T>A	p.Met182Lys	rs779728902		missense variant	T (0.35)	B (0.01)	23.9	1321453	0.005%
chr1:98165063	G	A	Het	1	1	0.02%	c.524C>T	p.Ser175Leu	rs371792178		missense variant	T (0.06)	B (0)	18.2	NA	0.004%
chr1:98187121	T	G	Het	1	0	0.02%	c.428A>C	p.Tyr143Ser	NA		missense variant	T (0.39)	B (0.08)	17.8	NA	
chr1:98205947	C	T	Het	1	0	0.02%	c.321+1G>A	p.(?)	rs746368304		splice donor variant			35	2439928	0.002%
chr1:98205966	N_1_	G	Het	3	1 (33%)	0.05%	c.299_302del	p.Phe100Serfs*15	rs539032572	*7	frameshift variant			33	495550	0.010%
chr1:98205983	C	G	Het	1	0	0.02%	c.286G>C	p.Asp96His	rs773159364		missense variant	D (0)	PD (0.99)	25.8	NA	<0.001%
chr1:98293727	A	N_2_	Het	1	0	0.02%	c.168_175dup	p.Phe59*	NA		stop gain				NA	
chr1:98348881	G	T	Het	1	1	0.02%	c.89C>A	p.Ser30Tyr	NA		missense variant	D (0)	PD (0.98)	26.9	NA	
chr1:98386443	G	A	Het	1	0	0.02%	c.36C>T	p.Ile12Ile	NA		synonymous variant			15.25	NA	
chr1:98386447	T	G	Het	1	0	0.02%	c.32A>C	p.Asp11Ala	NA		missense variant	D (0)	PD (0.84)	23.9	NA	
chr1:98386452	C	A	Het	1	0	0.02%	c.27G>T	p.Ser9Ser	NA		synonymous variant			13.9	NA	
chr1:98386478	T	C	Het	1	0	0.02%	c.1A>G	p.Met1?	rs772950053		start lost	D (0)	B (0.23)	24	NA	<0.001%

*Alt* alternative allele, *B* benign, *CADD* Combined Annotation Dependent Depletion, *D* deleterious, *Eur* european, *Freq* frequency, *Het* heterozygous, *Hom* homozygous, *MAF* minor allelic frequency, *N1* GATGA, *N2* AAATTATTC, *NA* not attributed, *PD* probably damaging, *Ref* reference allele, *T* tolerated.

### Association between the most clinically relevant *DPYD* defective variants and DPD deficiency

**Fig. 1:**
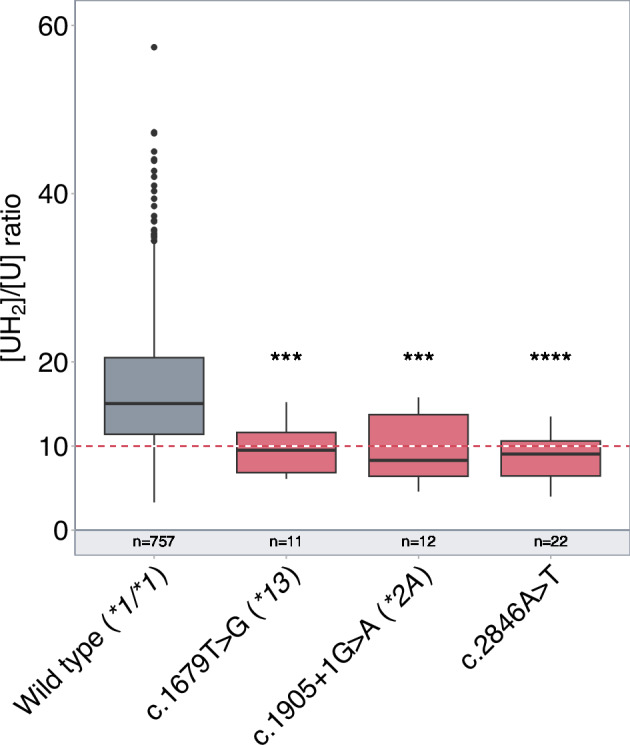
Association between the most clinically relevant *DPYD* defective rare variants and DPD deficiency. Box plot showing DPD pretreatment activity assessed by the dihydrouracil/uracil ([UH_2_]/[U]) plasma ratio according to the patient genotype. The box represents the 25–75% quartiles, the line in the box represents the median, whiskers represent the range. The red dash line indicates the ratio threshold used to categorize patients as having partial DPD deficiency (ratio ≤10) or normal DPD activity (ratio>10). *n* = number of patients; ***P < 0.001; ****P < 0.0001.

Dose adjustment based on pretreatment screening for the most clinically relevant *DPYD* defective variants, i.e. c.1679T>G (DPYD*13, rs55886062), c.1905+1G>A (DPYD*2A, rs3918290) and c.2846A>T (p.Asp949Val or rs67376798), has been shown to improve the safety of chemotherapy regimens based on fluorouracil [29]. Accordingly, international recommendations now provide indications for drug-related genetic tests and *DPYD* genotype-guided dosing in routine clinical practice [17, 18]. As expected, our data showed a significant association between each of these genetic variants and low DPD activity (Fig. 1).

### Association between common *DPYD* genetic variants and DPD deficiency

**Fig. 2:**
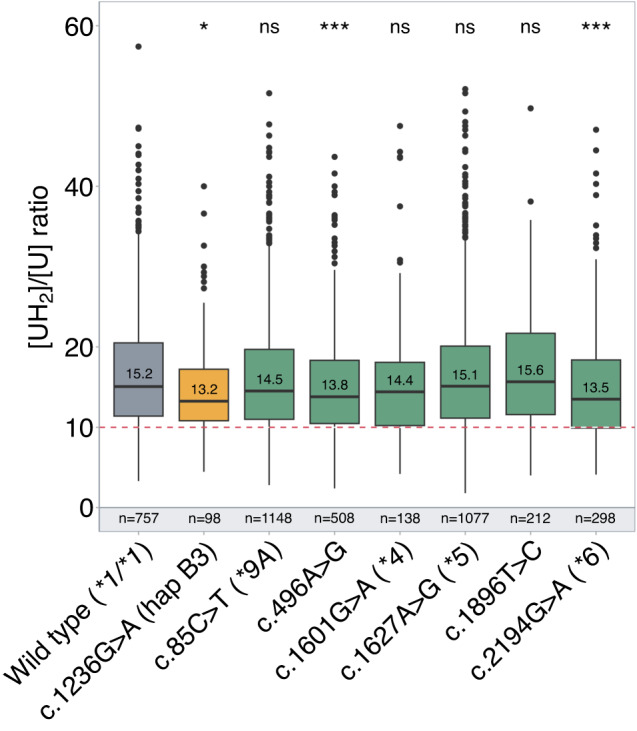
Association between common *DPYD* genetic variants and DPD deficiency. Box plot showing DPD pretreatment activity assessed by the dihydrouracil/uracil ([UH_2_]/[U]) plasma ratio according to the patient genotype. The hapB3 haplotype is represented in yellow whereas the other common variants are in green. The box represents the 25–75% quartiles, the line in the box represents the median, whiskers represent the range. The red dash line indicates the ratio threshold used to categorize patients as having partial DPD deficiency (ratio ≤10) or normal DPD activity (ratio >10). *n* = number of patients, ns = non-significant; *P < 0.05, **P < 0.01.

The association between common *DPYD* genetic variants (MAF ≥ 1%) and DPD activity is summarized in Fig. 2. Among the seven genetic variants identified, three variants (c.1236G>A or rs56038477 p.Glu412Glu ; c.496A>G or rs2297595 p.Met166Val; DPYD*6 c.2194G>A or rs1801160 p.Val732Ile) were significantly more frequent in the group of patients exhibiting partial DPD deficiency. Consistent with previous reports, the c.1236G>A (rs56038477) which is included in the risk haplotype B3 was significantly associated with low DPD activity [30, 31]. Nevertheless, compared to the most clinically relevant *DPYD* defective variants, the association of these three variants with DPD activity was rather modest (Fig. 2).

### Association between rare, very rare and novel *DPYD* genetic variants and DPD deficiency

**Fig. 3:**
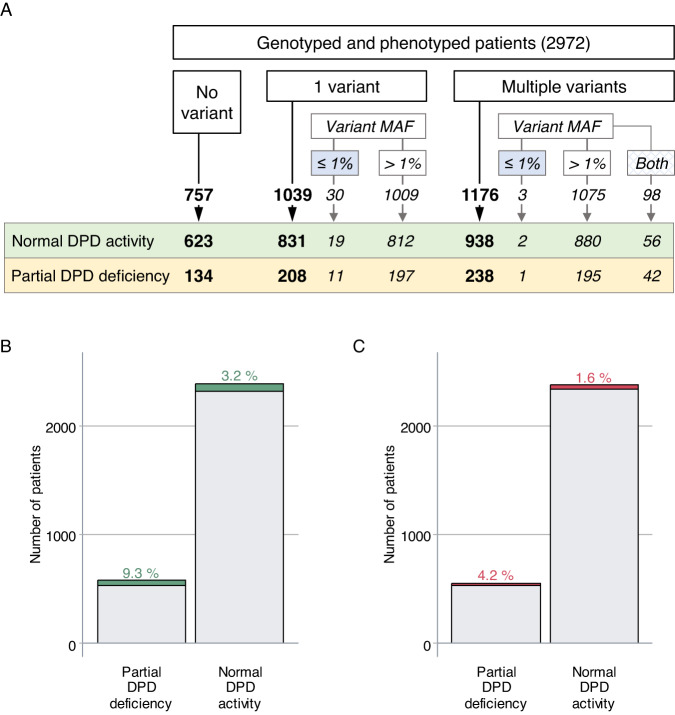
Association between *DPYD* genetic variant frequency and pretreatment DPD activity. (A) Flow chart showing the distribution of all identified *DPYD* genetic variants according to the minor allele frequency (MAF) in the groups of partial DPD deficiency ([UH_2_]/[U] plasma ratio below or equal to 10) and normal DPD activity ([UH_2_]/[U] plasma ratio above 10) (number of patients are reported) (B) Distribution of *DPYD* genetic variants based on minor allele frequency (MAF) below 1% according to pretreatment DPD activity (number of patients and percentage are reported). (C) Distribution of the *DPYD* genetic variants with a MAF below 1% and predicted to impact DPD activity (CADD score > 15) in the group of patients exhibiting normal or low DPD activity (number of patients and percentage are reported).

The list of frequent (MAF ≥ 1%), rare (MAF < 1%) and very rare (MAF ≤ 0.1%) variants identified in the *DPYD* gene in the whole cohort is summarized in Table 2. The number of patients in each group is summarized in Fig. 3A. Variants with a MAF below 1% were found to be enriched in patients exhibiting low DPD activity (9.3% versus 3.2% ; *P* < 0.00001) **(**Fig. 3B). This remained significant when excluding the rare clinically relevant *DPYD* defective variants (4.5% versus 2.6% ; *P* < 0.03). As many rare variants are likely to have little to no impact on DPD activity, a similar analysis including variants with a MAF below 1% and a putative deleterious impact on *DPYD* function according to CADD score (threshold above 15) was performed after excluding the rare clinically relevant *DPYD* defective variants. Indeed, a CADD score above 15 has been previously shown as a good prediction tool for pharmacogenetic variants [32]. Not surprisingly, these were more common in the group of patients with low DPD activity (4.2% versus 1.6% ; *P* < 0.001) (Fig. 3C). Overall, our results indicate that rare *DPYD* genetic variants account for a significative part of the interindividual variability of DPD activity.

## Discussion

Innovative and collaborative research efforts over the last decades have substantially improved our understanding of the role played by inherited genetic changes on the interindividual variability in drug efficacy or toxicity [33]. Large scale sequencing studies have notably shown that single-nucleotide variants are the most common form of protein-altering “functional variants” identified among genes relevant to the drug pharmacokinetics and pharmacodynamics, also known as pharmacogenes [33, 34]. Of particular interest, results from these studies have also revealed that rare genetic variants account for a substantial part of the unexplained interindividual differences in drug response, but their exact contribution on drug pharmacokinetics has not been systematically evaluated and remains thus poorly understood [33–36]. In this study, we focused on dihydropyrimidine dehydrogenase, a key enzyme in the metabolic catabolism of the chemotherapeutic agent 5-FU or its prodrugs, whose complete deficiency is associated with impaired clearance of 5-FU, excessive drug accumulation and severe toxicity.

Various genotyping and phenotyping approaches have been developed to assess DPD deficiency in order to reduce the incidence of severe toxicity without affecting treatment efficacy by dose tailoring fluoropyrimidine-based therapy. Although various uracil-based methods are routinely used in various countries to predict DPD deficiency, clinical relevance of pretreatment DPD phenotyping by these assays remains controversial [30]. Indeed, optimal cutoff levels that predict toxicity have not been validated yet and previous studies have shown extensive variability in uracil measurements when different cohorts were compared [12, 18, 37, 38]. In line with this, de With et al. [39]. very recently raised important issues against the utility of uracil-based assays in clinical practice given the large inter-center variability observed in measured pretreatment uracil levels. By contrast, the clinical validity of genotype-based approaches has been established in multiple metaanalyses as well as in large prospective studies [39, 40]. Results from these studies have in particular shown that prediction of DPD enzyme activity by molecular genetic testing in routine clinical practice is a reliable method that not only significantly improves patient safety but is also cost‐effective [41]. Consequently, clinical practice guidelines now recommend pre-emptive *DPYD* genotyping especially in Europe, where these four *DPYD* deficient alleles are relatively common in individuals of Caucasian ancestry [42]. Nevertheless, even using this strategy, prediction of fluoropyrimidine-induced toxicity remains suboptimal to detect all patients at risk of toxicity [43]. In this context, we aimed to assess whether rare genetic variants significantly contribute to the large interindividual variability of DPD enzyme characterizing a series of about 3 000 patients using new sequencing technologies.

Next Generation Sequencing (NGS) refers to a wide range of technologies enabling rapid and high-throughput sequencing of DNA [44]. In recent years, NGS has been successfully used to comprehensively interrogate the entire spectrum of genomic variations in pharmacogenes including rare variants [33]. In line with this, we applied an NGS-based approach to capture rare and common genetic variations located either in the coding sequence of the *DPYD* gene or its flanking intronic regions. Specifically, our results confirmed the strong impact of the three clinically rare variants. Additionally, although a significant association between DPD activity and three common known variants including Haplotype B3 was also shown in our large series of patients, their modest effect on DPD activity raises the question of their clinical relevance. Therefore, we suggest additional studies to clarify their use in prospective *DPYD* genotyping, especially as our study may be biased by several confounding factors. Of particular interest, our results also showed the importance of considering rare *DPYD* genetic variants to predict the risk of 5-FU toxicity. This is in agreement with results from sequencing data established in large distinct populations, which showed that the vast majority of variants among pharmacogenes are rare (MAF < 1%) or very rare (MAF ≤ 0.1%) and non-synonymous, with an estimated 30-40% of functional variability likely attributed to these rare variants [45]. For example, resequencing of 202 drug target genes in about 14 000 individuals showed that more than 95% of the identified variants had a MAF below 0.5% and that 90% of those were not known [46]. In light of our results, we suggest that additional studies should be performed to assess the association between rare *DPYD* genetic variants and fluoropyrimidine toxicity. This point is indeed of importance and represents one limitation of our study, as we could only assess the relationship between rare genetic variants and DPD activity.

In conclusion, our results strongly suggest that integrating rare genetic variants into routine pharmacogenetic testing can significantly improve the prediction of DPD enzyme activity. Therefore, we advocate that pre-emptive screening of DPD deficiency should be based on a more comprehensive genotyping approach, combined with phenotyping strategies, to ensure the safe administration of fluoropyrimidines.

## Supplementary information

Table S1. Demographic parameters of the whole cohort compared in male versus female patients.

Table S2. Custom-designed primer pairs and Sanger primer pairs.

Table S3. Hardy-Weinberg equilibrium and Minor Allele Frequency observed compared to GnomAD European frequencies for common (MAF ≥ 1) DPYD variants.

## Data Availability

Data and results are available at the Unit of Pharmacogenetics, University Hospital of Lille.

## References

[1] Relling MV, Klein TE, Gammal RS, Whirl-Carrillo M, Hoffman JM, Caudle KE. The clinical pharmacogenetics implementation consortium: 10 years later. Clin Pharm Ther. 2020;107:171–5. 10.1002/cpt.1651 PMC6925644 31562822

[2] Relling MV, Klein TE. CPIC: clinical pharmacogenetics implementation consortium of the pharmacogenomics research network. Clin Pharm Ther. 2011;89:464–7. 10.1038/clpt.2010.279 PMC3098762 21270786

[3] Swen JJ, van der Wouden CH, Manson LE, Abdullah-Koolmees H, Blagec K, Blagus T, et al. A 12-gene pharmacogenetic panel to prevent adverse drug reactions: an open-label, multicentre, controlled, cluster-randomised crossover implementation study. Lancet Lond Engl. 2023;401:347–56. 10.1016/S0140-6736(22)01841-4 36739136

[4] Rigter T, Jansen ME, de Groot JM, Janssen SWJ, Rodenburg W, Cornel MC. Implementation of pharmacogenetics in primary care: a multi-stakeholder perspective. Front Genet. 2020;11:10. 32076434 10.3389/fgene.2020.00010 PMC7006602

[5] St Sauver JL, Bielinski SJ, Olson JE, Bell EJ, Mc Gree ME, Jacobson DJ, et al. Integrating pharmacogenomics into clinical practice: promise vs reality. Am J Med. 2016;129:1093–1099.e1. Oct 27155109 10.1016/j.amjmed.2016.04.009 PMC5600492

[6] Haga SB, Kantor A. Horizon scan of clinical laboratories offering pharmacogenetic testing. Health Aff Proj Hope. 2018;37:717–23. 10.1377/hlthaff.2017.1564 PMC6642061 29733708

[7] Oates JT, Lopez D. Pharmacogenetics: an important part of drug development with a focus on its application. Int J Biomed Investig. 2018;1:111. 32467882 10.31531/2581-4745.1000111 PMC7255432

[8] Pasternak AL, Ward KM, Ateya MB, Choe HM, Thompson AN, Clark JS, et al. Establishment of a pharmacogenetics service focused on optimizing existing pharmacogenetic testing at a large academic health center. J Pers Med. 2020;10:154. 33023029 10.3390/jpm10040154 PMC7711716

[9] Evans WE, Relling MV. Moving towards individualized medicine with pharmacogenomics. Nature. 2004;429:464–8. 15164072 10.1038/nature02626

[10] Hulot JS, Bura A, Villard E, Azizi M, Remones V, Goyenvalle C, et al. Cytochrome P450 2C19 loss-of-function polymorphism is a major determinant of clopidogrel responsiveness in healthy subjects. Blood. 2006;108:2244–7. 16772608 10.1182/blood-2006-04-013052

[11] Yates CR, Krynetski EY, Loennechen T, Fessing MY, Tai HL, Pui CH, et al. Molecular diagnosis of thiopurine S-methyltransferase deficiency: genetic basis for azathioprine and mercaptopurine intolerance. Ann Intern Med. 1997;126:608–14. 9103127 10.7326/0003-4819-126-8-199704150-00003

[12] Knikman JE, Gelderblom H, Beijnen JH, Cats A, Guchelaar HJ, Henricks LM. Individualized dosing of fluoropyrimidine-based chemotherapy to prevent severe fluoropyrimidine-related toxicity: what are the options? Clin Pharm Ther. 2021;109:591–604. 10.1002/cpt.2069 PMC7983939 33020924

[13] Rosmarin D, Palles C, Church D, Domingo E, Jones A, Johnstone E, et al. Genetic markers of toxicity from capecitabine and other fluorouracil-based regimens: investigation in the QUASAR2 study, systematic review, and meta-analysis. J Clin Oncol J Am Soc Clin Oncol. 2014;32:1031–9. 10.1200/JCO.2013.51.1857 PMC4879695 24590654

[14] Meulendijks D, Henricks LM, Jacobs BAW, Aliev A, Deenen MJ, de Vries N, et al. Pretreatment serum uracil concentration as a predictor of severe and fatal fluoropyrimidine-associated toxicity. Br J Cancer. 2017;116:1415–24. 28427087 10.1038/bjc.2017.94 PMC5520099

[15] Pallet N, Hamdane S, Garinet S, Blons H, Zaanan A, Paillaud E, et al. A comprehensive population-based study comparing the phenotype and genotype in a pretherapeutic screen of dihydropyrimidine dehydrogenase deficiency. Br J Cancer. 2020;123:811–8. 32595208 10.1038/s41416-020-0962-z PMC7462856

[16] Schneider JJ, Galettis P, Martin JH. Overcoming barriers to implementing precision dosing with 5-fluorouracil and capecitabine. Br J Clin Pharm. 2021;87:317–25. 10.1111/bcp.14723 33386659

[17] Henricks LM, Lunenburg CATC, de Man FM, Meulendijks D, Frederix GWJ, Kienhuis E, et al. DPYD genotype-guided dose individualisation of fluoropyrimidine therapy in patients with cancer: a prospective safety analysis. Lancet Oncol. 2018;19:1459–67. 30348537 10.1016/S1470-2045(18)30686-7

[18] Diasio RB, Offer SM. Testing for dihydropyrimidine dehydrogenase deficiency to individualize 5-fluorouracil therapy. Cancers. 2022;14:3207. 35804978 10.3390/cancers14133207 PMC9264755

[19] Coudoré F, Roche D, Lefeuvre S, Faussot D, Billaud EM, Loriot MA, et al. Validation of an ultra-high performance liquid chromatography tandem mass spectrometric method for quantifying uracil and 5,6-dihydrouracil in human plasma. J Chromatogr Sci. 2012;50:877–84. 22689904 10.1093/chromsci/bms085

[20] Kristensen MH, Pedersen P, Mejer J. The value of dihydrouracil/uracil plasma ratios in predicting 5-fluorouracil-related toxicity in colorectal cancer patients. J Int Med Res. 2010;38:1313–23. 20926004 10.1177/147323001003800413

[21] Gamelin E, Boisdron-Celle M, Guérin-Meyer V, Delva R, Lortholary A, Genevieve F, et al. Correlation between uracil and dihydrouracil plasma ratio, fluorouracil (5-FU) pharmacokinetic parameters, and tolerance in patients with advanced colorectal cancer: a potential interest for predicting 5-FU toxicity and determining optimal 5-FU dosage. J Clin Oncol J Am Soc Clin Oncol. 1999;17:1105. 10.1200/JCO.1999.17.4.1105 10561167

[22] Jiang H, Lu J, Jiang J, Hu P. Important role of the dihydrouracil/uracil ratio in marked interpatient variations of fluoropyrimidine pharmacokinetics and pharmacodynamics. J Clin Pharm. 2004;44:1260–72. 10.1177/0091270004268911 15496644

[23] Zhou ZW, Wang GQ, Wan DS, Lu ZH, Chen YB, Li S, et al. The dihydrouracil/uracil ratios in plasma and toxicities of 5-fluorouracil-based adjuvant chemotherapy in colorectal cancer patients. Chemotherapy. 2007;53:127–31. 17308379 10.1159/000099984

[24] Wettergren Y, Carlsson G, Odin E, Gustavsson B. Pretherapeutic uracil and dihydrouracil levels of colorectal cancer patients are associated with sex and toxic side effects during adjuvant 5-fluorouracil-based chemotherapy. Cancer. 2012;118:2935–43. 22020693 10.1002/cncr.26595

[25] Mueller F, Büchel B, Köberle D, Schürch S, Pfister B, Krähenbühl S, et al. Gender-specific elimination of continuous-infusional 5-fluorouracil in patients with gastrointestinal malignancies: results from a prospective population pharmacokinetic study. Cancer Chemother Pharm. 2013;71:361–70. 10.1007/s00280-012-2018-4 23139054

[26] Galarza AFA, Linden R, Antunes MV, Hahn RZ, Raymundo S, da Silva ACC, et al. Endogenous plasma and salivary uracil to dihydrouracil ratios and DPYD genotyping as predictors of severe fluoropyrimidine toxicity in patients with gastrointestinal malignancies. Clin Biochem. 2016;49:1221–6. 27399164 10.1016/j.clinbiochem.2016.07.004

[27] Boisdron-Celle M, Remaud G, Traore S, Poirier AL, Gamelin L, Morel A, et al. 5-Fluorouracil-related severe toxicity: a comparison of different methods for the pretherapeutic detection of dihydropyrimidine dehydrogenase deficiency. Cancer Lett. 2007;249:271–82. 17064846 10.1016/j.canlet.2006.09.006

[28] McKenna A, Hanna M, Banks E, Sivachenko A, Cibulskis K, Kernytsky A, et al. The Genome Analysis Toolkit: a MapReduce framework for analyzing next-generation DNA sequencing data. Genome Res. 2010;20:1297–303. 20644199 10.1101/gr.107524.110 PMC2928508

[29] Wigle TJ, Povitz BL, Medwid S, Teft WA, Legan RM, Lenehan J, et al. Impact of pretreatment dihydropyrimidine dehydrogenase genotype-guided fluoropyrimidine dosing on chemotherapy associated adverse events. Clin Transl Sci. 2021;14:1338–48. 33620159 10.1111/cts.12981 PMC8301551

[30] van Kuilenburg ABP, Meijer J, Mul ANPM, Meinsma R, Schmid V, Dobritzsch D, et al. Intragenic deletions and a deep intronic mutation affecting pre-mRNA splicing in the dihydropyrimidine dehydrogenase gene as novel mechanisms causing 5-fluorouracil toxicity. Hum Genet. 2010;128:529–38. 20803296 10.1007/s00439-010-0879-3 PMC2955237

[31] Meulendijks D, Henricks LM, van Kuilenburg ABP, Jacobs BAW, Aliev A, Rozeman L, et al. Patients homozygous for DPYD c.1129-5923C>G/haplotype B3 have partial DPD deficiency and require a dose reduction when treated with fluoropyrimidines. Cancer Chemother Pharm. 2016;78:875–80. 10.1007/s00280-016-3137-0 27544765

[32] Zhou Y, Mkrtchian S, Kumondai M, Hiratsuka M, Lauschke VM. An optimized prediction framework to assess the functional impact of pharmacogenetic variants. Pharmacogenomics J. 2019;19:115–26. 30206299 10.1038/s41397-018-0044-2 PMC6462826

[33] Schwarz UI, Gulilat M, Kim RB. The role of next-generation sequencing in pharmacogenetics and pharmacogenomics. Cold Spring Harb Perspect Med. 2019;9:a033027. 29844222 10.1101/cshperspect.a033027 PMC6360866

[34] Zhou Y, Tremmel R, Schaeffeler E, Schwab M, Lauschke VM. Challenges and opportunities associated with rare-variant pharmacogenomics. Trends Pharm Sci. 2022;43:852–65. 36008164 10.1016/j.tips.2022.07.002

[35] Ingelman-Sundberg M, Mkrtchian S, Zhou Y, Lauschke VM. Integrating rare genetic variants into pharmacogenetic drug response predictions. Hum Genomics. 2018;12:26. 29793534 10.1186/s40246-018-0157-3 PMC5968569

[36] Callon S, Brugel M, Botsen D, Royer B, Slimano F, Feliu C, et al. Renal impairment and abnormal liver function tests in pre-therapeutic phenotype-based DPD deficiency screening using uracilemia: a comprehensive population-based study in 1138 patients. Ther Adv Med Oncol. 2023;15:17588359221148536. 36643657 10.1177/17588359221148536 PMC9837271

[37] Deenen MJ, Meulendijks D, Cats A, Sechterberger MK, Severens JL, Boot H, et al. Upfront genotyping of DPYD*2A to individualize fluoropyrimidine therapy: a safety and cost analysis. J Clin Oncol J Am Soc Clin Oncol 2016;34:227–34. Jan 20 10.1200/JCO.2015.63.1325 26573078

[38] Sistonen J, Büchel B, Froehlich TK, Kummer D, Fontana S, Joerger M, et al. Predicting 5-fluorouracil toxicity: DPD genotype and 5,6-dihydrouracil:uracil ratio. Pharmacogenomics. 2014;15:1653–66. 25410891 10.2217/pgs.14.126

[39] de With M, Knikman J, de Man FM, Lunenburg CATC, Henricks LM, van Kuilenburg ABP, et al. Dihydropyrimidine dehydrogenase phenotyping using pretreatment uracil: a note of caution based on a large prospective clinical study. Clin Pharm Ther. 2022;112:62–8. 10.1002/cpt.2608 PMC9322339 35397172

[40] Henricks LM, Opdam FL, Beijnen JH, Cats A, Schellens JHM. DPYD genotype-guided dose individualization to improve patient safety of fluoropyrimidine therapy: call for a drug label update. Ann Oncol J Eur Soc Med Oncol. 2017;28:2915–22. 10.1093/annonc/mdx411 29045513

[41] Froehlich TK, Amstutz U, Aebi S, Joerger M, Largiadèr CR. Clinical importance of risk variants in the dihydropyrimidine dehydrogenase gene for the prediction of early-onset fluoropyrimidine toxicity. Int J Cancer. 2015;136:730–9. 24923815 10.1002/ijc.29025

[42] Paulsen NH, Pfeiffer P, Ewertz M, Fruekilde PBN, Feddersen S, Holm HS, et al. Implementation and clinical benefit of DPYD genotyping in a Danish cancer population. ESMO Open. 2023;8:100782. 36791638 10.1016/j.esmoop.2023.100782 PMC10024141

[43] Rare genetic variant burden in DPYD predicts severe fluorop, De Mattia E, Silvestri M, Polesel J, Ecca F, Mezzalira S, Scarabel L, et al. yrimidine-related toxicity risk. Biomed Pharmacother Biomed Pharmacother. 2022;154:113644. 36063648 10.1016/j.biopha.2022.113644 PMC9463069

[44] Larrue R, Chamley P, Bardyn T, Lionet A, Gnemmi V, Cauffiez C, et al. Diagnostic utility of whole-genome sequencing for nephronophthisis. NPJ Genom Med. 2020;5:38. 33024573 10.1038/s41525-020-00147-8 PMC7506526

[45] Kozyra M, Ingelman-Sundberg M, Lauschke VM. Rare genetic variants in cellular transporters, metabolic enzymes, and nuclear receptors can be important determinants of interindividual differences in drug response. Genet Med J Am Coll Med Genet. 2017;19:20–9. 10.1038/gim.2016.33 27101133

[46] Nelson MR, Wegmann D, Ehm MG, Kessner D, St Jean P, Verzilli C, et al. An abundance of rare functional variants in 202 drug target genes sequenced in 14,002 people. Science. 2012;337:100–4. 22604722 10.1126/science.1217876 PMC4319976

