# Correction: Integrating rare genetic variants into *DPYD* pharmacogenetic testing may help preventing fluoropyrimidine-induced toxicity

**DOI:** 10.1038/s41397-025-00381-2

**Published:** 2025-07-15

**Authors:** Romain Larrue, Sandy Fellah, Benjamin Hennart, Naoual Sabaouni, Nihad Boukrout, Cynthia Van der Hauwaert, Clément Delage, Meyling Cheok, Michaël Perrais, Christelle Cauffiez, Delphine Allorge, Nicolas Pottier

**Affiliations:** 1 Univ. Lille, CNRS, Inserm, CHU Lille, Institut Pasteur de Lille, UMR9020-U1277-CANTHER-Cancer Heterogeneity Plasticity and Resistance to Therapies, F-59000 Lille, France; 2 Service de Toxicologie et Génopathies, CHU Lille, F-59000 Lille, France

**Keywords:** Predictive markers, Genetic testing

Correction to: *The Pharmacogenomics Journal* (2023) **24**:1 10.1038/s41397-023-00322-x, published online 12 January 2024

In Fig. 1 of this article; the figure was given as the 1896 mutation, but should have appeared as the 2846 mutation.

Original Figure


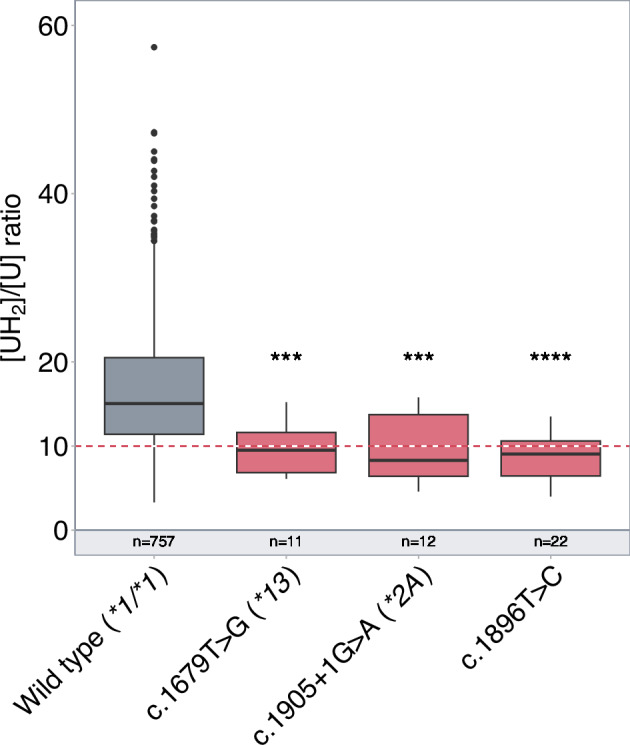


Corrected Figure


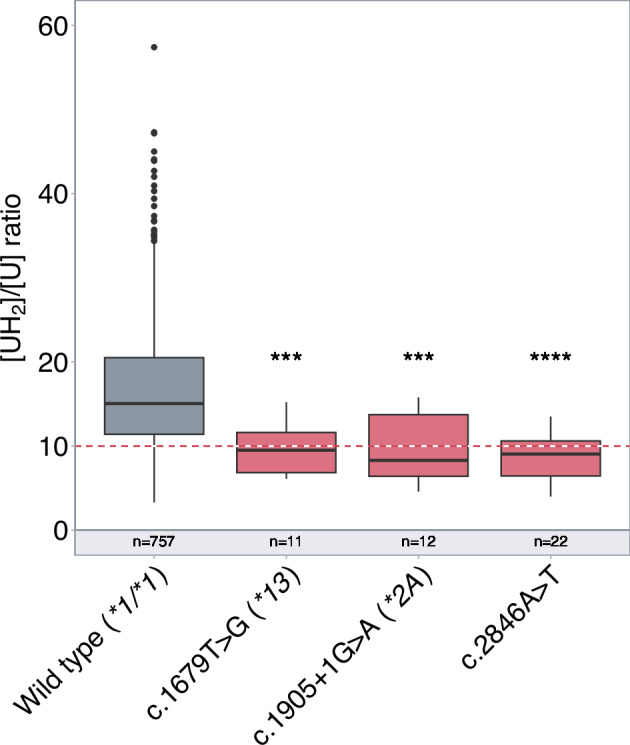


The original article has been corrected.

